# SPATIAL NAVIGATION IN ALZHEIMER’S DISEASE - A META-ANALYSIS

**DOI:** 10.1093/geroni/igae098.3723

**Published:** 2024-12-31

**Authors:** Dorota Kossowska-Kuhn, Gillian Gouveia, Neil Charness, Richard Wagner

**Affiliations:** Florida State University, Tallahassee, Florida, United States; Florida State University, Tallahassee, Florida, United States; Weill Cornell Medicine, New York, New York, United States; Florida State University, Tallahassee, Florida, United States

## Abstract

The global population of individuals aged 65 and over is projected to nearly double by 2050, reaching 1.6 billion. This demographic shift is expected to drive a significant increase in dementia cases, with Alzheimer’s disease (AD) accounting for 60-80% of these cases. While episodic memory loss is a hallmark of AD, spatial disorientation is often one of the earliest symptoms (Coughlan et al. 2018).This meta-analysis investigates the differences in spatial navigation performance between cognitively healthy older adults and those with AD. The effect size is measured using Hedge’s g. The analysis considers various moderators, including the mode of test administration (real-world, virtual reality, computer screen), the type of measure (time, accuracy), and the nature of the task (maze navigation, environmental navigation), alongside basic study characteristics such as publication year, study location, participant age, gender, and education.The analysis includes 124 effect sizes from 42 studies, involving 2409 participants, of which 941 were diagnosed with AD and 1468 were cognitively healthy older adults. The results show that cognitively healthy older adults have significantly better navigation abilities (p < 0.05) compared to those with AD, as indicated by a standardized mean difference (SMD) of 1.97.This study shows a significant decline in spatial navigation abilities in individuals with AD compared to healthy older adults, consistent across various test methods and tasks. Addressing these deficits is vital to reduce the growing impact of dementia.

